# Malaria parasites and related haemosporidians cause mortality in cranes: a study on the parasites diversity, prevalence and distribution in Beijing Zoo

**DOI:** 10.1186/s12936-018-2385-3

**Published:** 2018-06-18

**Authors:** Ting Jia, Xi Huang, Gediminas Valkiūnas, Minghai Yang, Changming Zheng, Tianchun Pu, Yanyun Zhang, Lu Dong, Xun Suo, Chenglin Zhang

**Affiliations:** 1 College of Veterinary Medicine, China Agricultural University, Beijing, 100193 China; 2 Beijing Key Laboratory of Captive Wildlife Technologies, Beijing Zoo, Beijing, 100044 China; 3 Department of Biology, Lund University, 22362 Lund, Sweden; 4 Nature Research Centre, Akademijos 2, 08412 Vilnius, Lithuania; 5 Ministry of Education Key Laboratory for Biodiversity and Ecological Engineering, College of Life Sciences, Beijing Normal University, Beijing, 100875 China

**Keywords:** Avian malaria, Haemosporidian parasite diversity, Captive crane, Mortality, Ex situ conservation

## Abstract

**Background:**

Malaria parasites and related haemosporidian parasites are widespread and may cause severe diseases in birds. These pathogens should be considered in projects aiming breeding of birds for purposes of sustained ex situ conservation. Cranes are the ‘flagship species’ for health assessment of wetland ecosystems, and the majority of species are endangered. Malaria parasites and other haemosporidians have been reported in cranes, but the host-parasite relationships remain insufficiently understood. Morbidity of cranes due to malaria has been reported in Beijing Zoo. This study report prevalence, diversity and distribution of malaria parasites and related haemosporidians in cranes in Beijing Zoo and suggest simple measures to protect vulnerable individuals.

**Methods:**

In all, 123 cranes (62 adults and 61 juveniles) belonging to 10 species were examined using PCR-based testing and microscopic examination of blood samples collected in 2007–2014. All birds were maintained in open-air aviaries, except for 19 chicks that were raised in a greenhouse with the aim to protect them from bites of blood-sucking insects. Bayesian phylogenetic analysis was used to identify the closely related avian haemosporidian parasites.

**Results:**

Species of *Plasmodium* (5 lineages), *Haemoproteu*s (1) and *Leucocytozoon* (2) were reported. Malaria parasites predominated (83% of all reported infections). The overall prevalence of haemosporidians in juveniles was approximately seven-fold higher than in adults, indicating high susceptibility of chicks and local transmission. Juvenile and adult birds hosted different lineages of *Plasmodium*, indicating that chicks got infection from non-parent birds. *Plasmodium relictum* (pSGS1) was the most prevalent malaria parasite. Mortality was not reported in adults, but 53% of infected chicks died, with reports of co-infection with *Plasmodium* and *Leucocytozoon* species. All chicks maintained in the greenhouse were non-infected and survived. Species of *Leucocytozoon* were undetectable by commonly used PCR protocol, but readily visible in blood films.

**Conclusion:**

Crane chicks often die due to malaria and *Leucocytozoon* infections, which they likely gain from wild free-living birds in Beijing Zoo. Molecular diagnostics of crane *Leucocytozoon* parasites needs improvement. Because the reported infections are mainly chick diseases, the authors recommend maintaining of juvenile birds in vector-free facilities until the age of approximately 6 months before they are placed in open-air aviaries.

**Electronic supplementary material:**

The online version of this article (10.1186/s12936-018-2385-3) contains supplementary material, which is available to authorized users.

## Background

Avian haemosporidian parasites (Haemosporida) of the genera *Plasmodium, Haemoproteus* and *Leucocytozoon* are widespread and can cause diseases in wild and domestic birds. Therefore, they have been subjects of much research [1–5]. These parasites are easy to sample and have been often used as model organisms in evolutionary biology studies [6]. During the past 15 years, much new information about genetic diversity of avian haemosporidians was collected [5, 7–10], and molecular characterization of many of these parasite species was developed, providing opportunities to improve infection diagnostics both in avian hosts and vectors [4, 8, 11–13]. Information about patterns of distribution of haemosporidians in wild birds is important for better understanding of opportunities for the long-distance transport of infections by migrating birds and the emergence of new parasitic infections [14]. Small wild birds, particularly belonging to Passeriformes are relatively well sampled for haemosporidian research because they are easy to catch using simple mist-netting methods [1], but large birds, particularly protected species, remain markedly under-sampled [7, 10]. Examination of birds in zoos and rehabilitation centres has drawbacks because these animals often placed at such sites unnaturally and may bring parasites and other disease agents with them thereby creating an unnatural community of organisms. However, parasitological studies in zoos and other centres usually are under professional veterinary control and provide valuable information about parasites. This is particularly valuable in case of large protected bird species, which are difficult to sample in the wild [15]. Scott was the first to publish information about severe diseases caused by haemosporidian parasites in captive zoo birds in London [16, 17], and severe haemosporidioses were reported subsequently in many zoos in Asia, Europe, South and North Americas and Africa [18–25]. Recent studies indicate potential epidemiological risks of malaria and other haemosporodioses in zoo birds [24–27]. Nevertheless, reports of haemosporidians remain relatively rare in captive birds, with only approximately 100 records of mainly unidentified haemosporidian species available in MalAvi database [7].

Cranes (Gruiformes) are known as ‘flagship species’ for health assessment of wetland ecosystems [28, 29]. Because the majority of gruiform birds are endangered and protected species [30], ex situ conservation projects were established in many countries aiming to keep these birds’ populations in good size and to support biodiversity conservation. Haemosporidian infections have been only occasionally reported in captive cranes (http://mbio-serv2.mbioekol.lu.se/Malavi/about.html) [16, 31–33], but these blood parasites might be obstacles for successful implementation of ex situ conservation projects [26].

Beijing Zoo maintains sustainable population of several endangered cranes species, including the black-necked crane *Grus nigricollis*, the red-crowned crane *Grus japonensis*, the white-naped crane *Grus vipio* and some other species. Haemosporidian infections were detected in cranes in this zoo [34]. This study analysed data about prevalence and diversity of avian haemosporidian parasites reported in Beijing Zoo cranes during an 8-year period of monitoring. Sources of haemosporidian infections were determined in cranes, parasite diversity was described, mortality was reported in chicks and preventive measures were suggested to minimize a probability of haemosporidiosis in captive zoo birds.

## Methods

### Study site, collection of blood samples and microscopic analysis

**Fig. 1:**
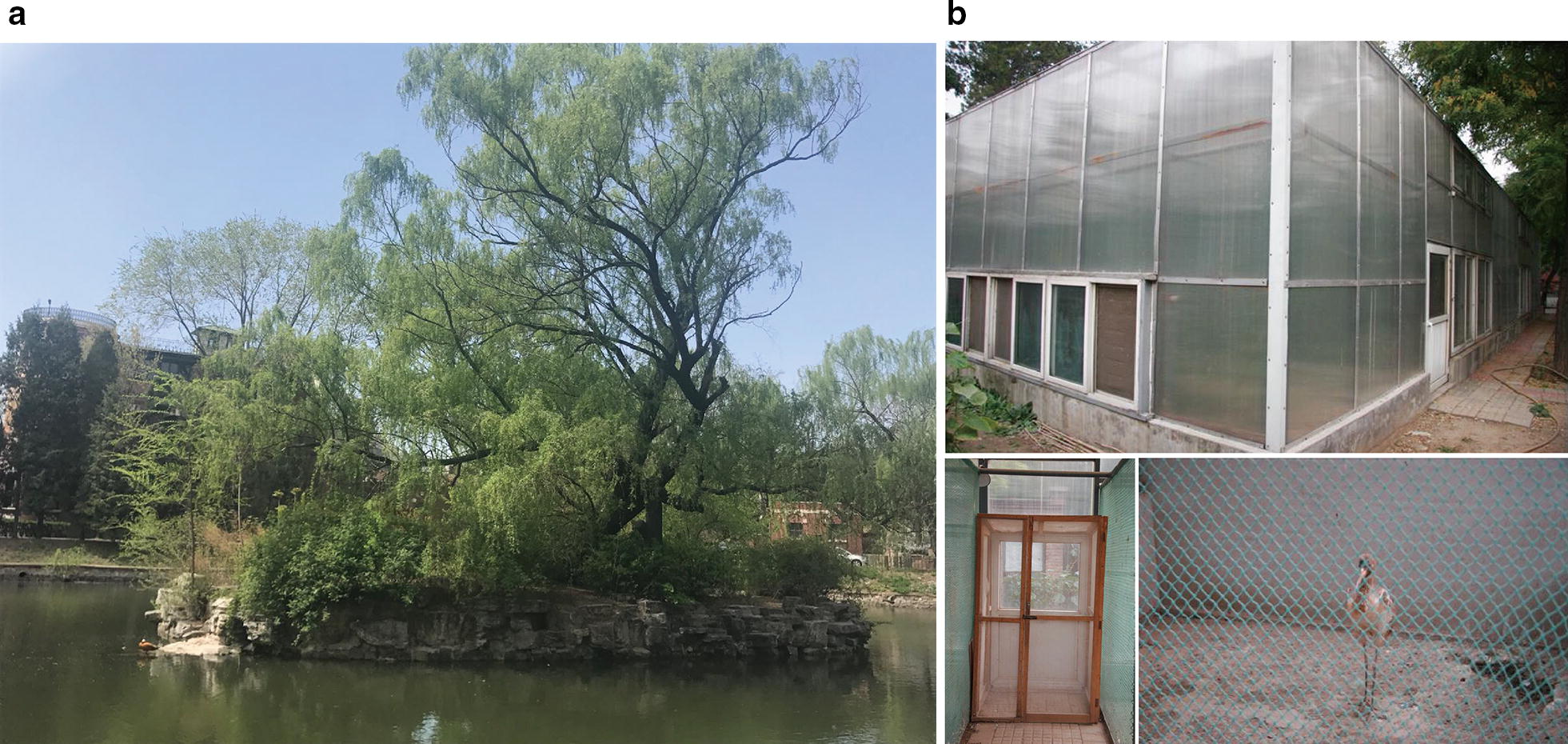
Two captive sites for cranes in the Beijing Zoo: Crane Island (**a**) and greenhouse (**b**). Explanations are given in the text

The cranes were maintained on a 8680-square-metre Crane Island (Beijing, China, N39°56′23.60″, E116°19′29.49″), which is located in the middle of a stagnant lake (Fig. 1a). The island is wooded, and its vegetation is mainly temperate tree, shrub and complex structure of grasses. Persimmon tree (*Diospyros* sp.), poplar (*Liriodendron* sp.), sycamore (*Firmiana platanifolia*), willow (*Salix alba*), Amur honeysuckle (*Lonicera maackii*) and other plants grow on this island. Crane adults and juveniles (aged between 1 and 52 week-old) were maintained together, and they were exposed to natural bites of blood-sucking insects. As the island was surrounded by water, many mosquitoes were reported on the island during the warm times of year, while no *Culicoides* biting midges or simuliid flies (*Simuliidae*) were observed. Cranes were mostly from three origins: rescue from natural habitat, introduction from other rehabilitation centres and artificial breeding in the Zoo.

In all, 123 individual birds (62 adults and 61 juveniles) belonging to 10 species were examined in 2007–2014 (Table 1). The majority of sampled birds were maintained on the Crane Island, but 19 chicks were kept in a greenhouse between 2008 and 2012 (Fig. 1b), which was built in the zoo with the aim to grow juvenile birds in the environment free of blood-sucking insects. The greenhouse was located in a dry and woodless area; it was equipped with ventilation system and the double-screened doors prevented penetration of blood-sucking insects in this facility.

**Table 1:** Prevalence and diversity of haemosporidian parasites in cranes maintained in open aviaries and examined by PCR diagnostics in Beijing Zoo, 2007–2014

Bird species	Adult						Juvenile					
	No. examined	No. positive	Infected with				No. examined	No. positive	Infected with			
			*Pl*	*H*	*L*	Lineage			*Pl*	*H*	*L*	Lineage
*Anthropodies paradisea*	9	0	0	0	0		4	1	1	0	0	C
*Balearica pavonina*	3	0	0	0	0		4	0	0	0	0	
*Balearica regulorum*	8	0	0	0	0		2	0	0	0	0	
*Bugeranus carunculatus*	0	0	0	0	0		2	1	1	0	0	C
*Grus antigone*	1	0	0	0	0		0	0	0	0	0	
*Grus japonensis*	25	1^2^	0	0	1^2^	G^1^	11	4	4	0	0	A
*Grus monacha*	0	0	0	0	0		1	1	1	0	0	D
*Grus leucogeranus*	4	1^3^	0	1^3^	0	F	4	2	2	0	0	A
*Grus nigricollis*	9	0	0	0	0		10	9	9	0	0	A^1^, E
*Grus vipio*	3	2^4^	1^a^	1^b^	1^c^	B, F, H	4	2	1	1	0	A, F
Total (10 species)	62	4 (6.5)	1 (1.6)	2 (3.2)	2 (3.2)	4 lineages	42	20 (47.6)	19 (45.2)	1 (2.4)	0	5 lineages

Pl—*Plasmodium*, H—*Haemoproteus*, L—*Leucocytozoon*. Prevalence of infection (in percentage) is given in parentheses. *Plasmodium* spp. lineages: A—pSGS1, B—p SW2, C—pANTPAR01, D—pGRUMON01, E—pGRUNIG01; *Haemoproteus* sp. lineage: F—hGRUVIP01; *Leucocytozoon* spp. lineages: G—lGRUJAP02, H—lCB1

^1^Indicate microscopy positive samples

^2^This *Grus japonensis* individual originated from artificial breeding in the Zoo was infected with lineage lGRUJAP02

^3^This *Grus leucogeranus* individual originated from Nanjing Red Forest Zoo was infected with lineage hGRUVIP01

^4^These 2 *Grus vipio* individuals were from natural habitat of northeastern China, (a) infected with lineage pSW2; (b) lineage hGRUVIP01 and (c) lCB1

Blood samples were collected between July and September in 2007–2014. Blood from all birds was taken from the metatarsal vein for microscopic examination and molecular analysis. Blood films were prepared immediately after withdrawal of the blood, fixed with methanol and stained with Giemsa [1]. Blood samples were also collected for molecular analysis (see below); they were stored in EDTA and maintained at − 80 °C approximately.

Five µl of fresh blood was dropped on a slide and smeared prepare to a thin blood film. At least two blood films were prepared from each bird. After methanol fixation, the slides were incubated with Giemsa stain (BA4107, Baso diagnostics Inc., Zhuhai, China) at room temperature for 1 h. After washing and natural drying, the stained blood films were examined microscopically. Approximately 100–150 fields were screened at low magnification (× 400) and then 100 fields were studied at high magnification (× 1000). An Olympus BX53 light microscope equipped with imaging software (cellSens Standard) was used in morphological analysis.

### Molecular analysis

Total DNA was extracted from blood samples using a TIANamp DNA kit (Tiangen, Beijing) according to the manufacturer’s instructions. A nested PCR protocol was applied for amplification of a 479 bp fragment of the mitochondrial cytochrome *b* gene (*cytb*) [35]. For the 1st PCR, the primers HaemNFI (5′-CATATATTAAGAGAAITATGGAG-3′) and HaemNR3 (5′-ATAGAAAGATAAGAAATACCATTC-3′) were used. In the 2nd PCR, two primer pairs were applied: the primers HaemNF (5′-ATGGTGCTTTCGATATATGCATG-3′) and HaemNR2 (5′-GCATTATCTGGATGTGATAATGGT-3′), and also HaemNFL (5′-ATGGTGTTTTAGATACTTACATT-3′) and HaemNR2L (5′-CATTATCTGGATGAGATAATGGIGC-3′). Amplification success was tested by running 2 μl of the 2nd PCR product on 1.5% agarose gel stained with SYBR Green I and visualisation in an ultraviolet trans-illuminator (GDS-8000PC, GENE, USA). One negative control (nuclease-free water) and three positive controls (one *Plasmodium* sample, one *Haemoproteus* sample, and one *Leucocytozoon* sample, which were positive by microscopic examination of blood films) were used to determine possible false amplifications. No case of false amplification was found.

**Fig. 2:**
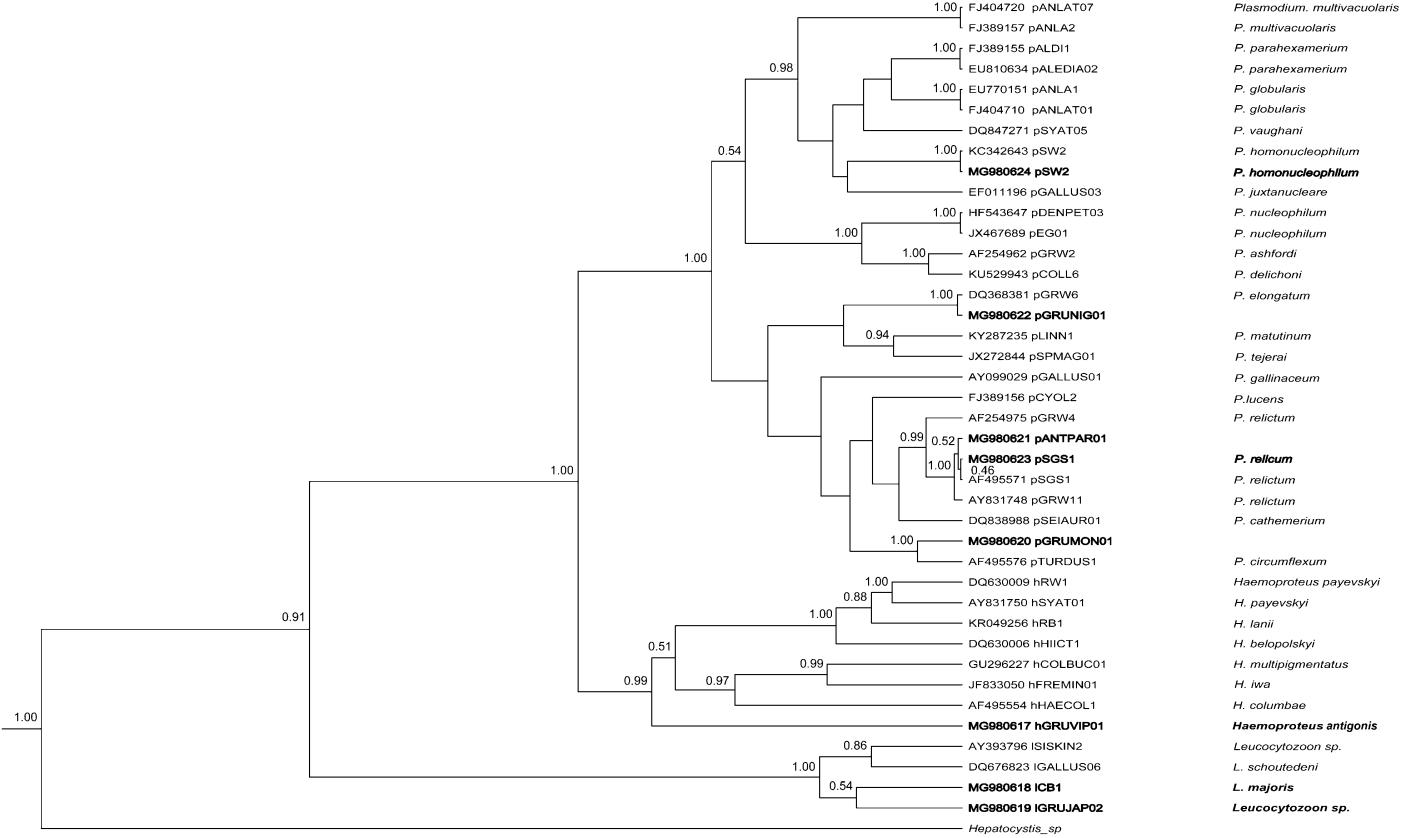
Bayesian phylogeny of 29 mitochondrial cytochrome *b* lineages of *Plasmodium* species, 8 lineages of *Haemoproteus* spp. and 4 lineages of *Leucocytozoon* spp. One lineage of *Hepatocystis* sp. was used as an outgroup. Codes of the lineages (according to MalAvi database, http://mbio-serv2.mbioekol.lu.se/Malavi), parasite species names and GenBank accession numbers are provided in the tree. Posterior probabilities of > 0.5 are indicated near the nodes. The branch lengths are drawn proportionally to the amount of change (scale bars are shown). The parasite lineages reported in this study are given Bold

The PCR products were purified and sequenced in both directions using a 3730XL automatic sequencer (ABI, USA). The sequences were assembled by CodonCode Aligner 5.1.5 (CodonCode Corporation, USA). Each sample was sequenced three times to check the repeatability of results. Sequences were identified by DnaSP 5.10.01 (Librado and Rozas, 2009) and then aligned in MEGA 5.04 together with most similar lineages according to the BLAST^®^ result in MalAvi database (http://mbio-serv2.mbioekol.lu.se/Malavi/blast.html) [7]. Haplotypes were defined as new lineages if they differ by 1 bp from lineages deposited in the MalAvi database (see http://mbio-serv2.mbioekol.lu.se/Malavi). A fragment of *cytb* sequence of *Hepatocystis* sp. (Genbank No. KC262867.1) was used as an outgroup for rooting due to its close genetic relationship with avian haemosporidians [9]. To address the phylogenetic relationships of the reported crane haemosporidians, 41 lineages of parasites belonging to different haemosporidian genera were included based on morphologically identified parasite species (Fig. 2). Nucleotide substitution models were tested using jModel Test 2.1.4 [36]. Best-fit model was determined using Akaike Information Criterion (AICc). Bayesian phylogenetic inference was constructed using BEAST v1.8.0 [37] with default parameters, as well as strict molecular clock and Yule process for tree prior. Markov chain Monte Carlo (MCMC) was run for different steps, with the length of the chain set as 1 × 10^9^ and log parameters as every 1 × 10^5^ generation. The maximum credibility tree was searched by Tree Annotator v1.8.0 after the first 1000 trees were discarded as burnin. The selected tree was then adjusted in software FigTree v1.3.1 (Andrew Rambaut, University of Edinburgh, UK; http://tree.bio.ed.ac.uk/software/figtree/). Visualization of “double bases” in electropherograms of sequences was used to estimate presence of possible haemosporidian co-infections [38]. Genetic differences between different lineages of *P. relictum* were calculated using the Jukes–Cantor model of substitution, as implemented in the programme MEGA 7.0 [39].

### Post-mortem examination of juvenile cranes

Interstitial fluid of liver and spleen from died chick were inoculated on blood agar plates, incubated at 37 °C overnight and analysed using a Microbial Identification and Antimicrobial System (BIOFOSUN-II, Shanghai Biofosun Biotech Co., LTD, Shanghai, China). Blood clots in cardiac chamber were sampled, and the blood serum was then separated by centrifugation for detection of the H5 subtype of avian influenza virus and Newcastle disease virus according to the manufacturer’s instructions (ADD THE NAME OF PROTOCOL, IF POSSIBLE). Organs were also examined for possible presence of helminths. For haemosporidian and other protozoan parasites detection, touch imprint of liver, spleen and lungs were prepared on glass slides, stained with Giemsa as described above, and examined microscopically. The saturated solution floating method was used for investigation of parasites in intestinal contents as well.

## Results

**Fig. 3:**
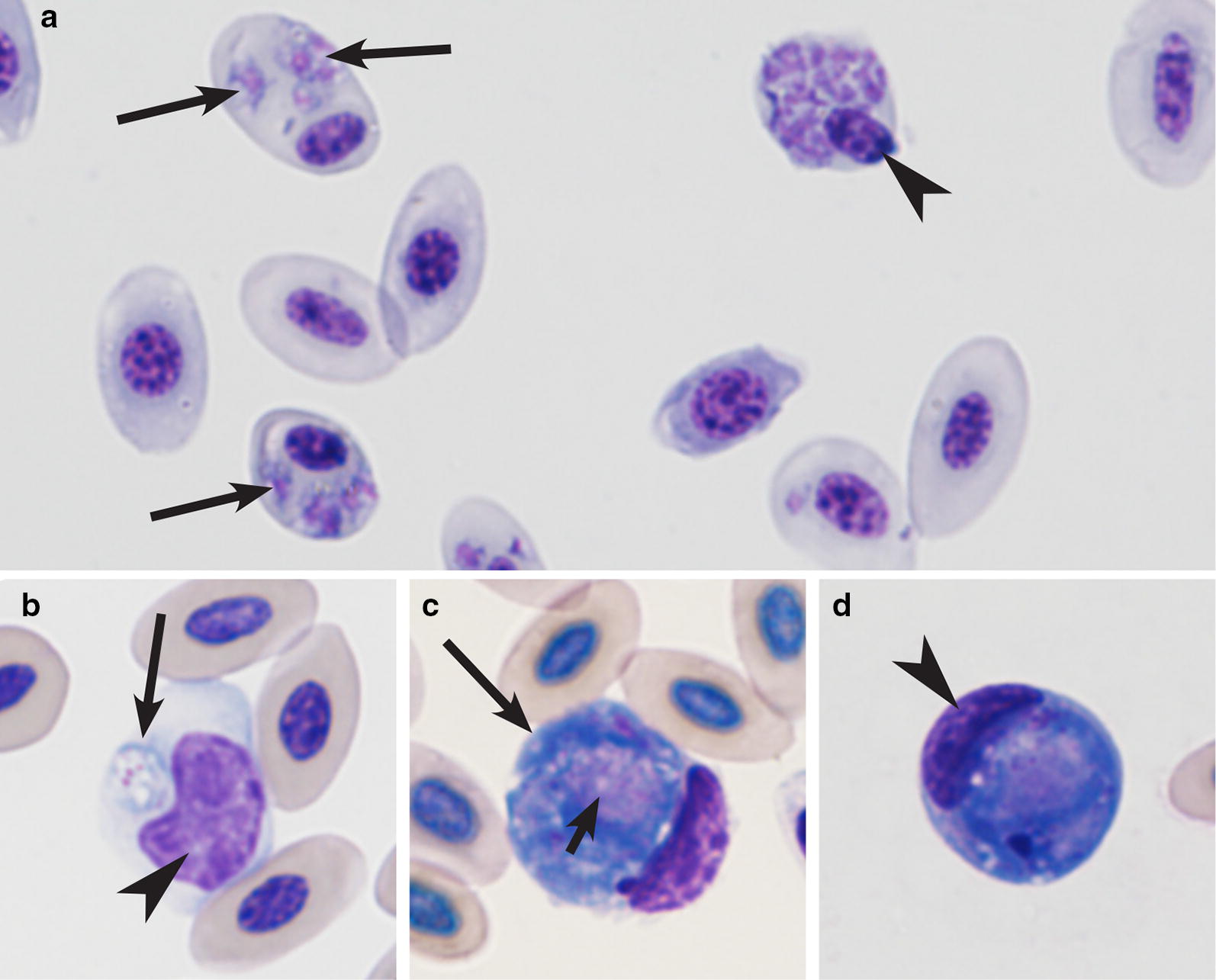
Blood stages of haemosporidian parasites reported in cranes in the Beijing Zoo: *Plasmodium relictum* (lineages pSGS1, **a**) and *Leucocytozoon* sp. (lineage in unknown, **b**–**d**): **a** heavy parasitaemia in Black-necked crane (*Grus nigricollis*) (note numerous developing trophozoites, meronts and multiple infections of erythrocytes); **b**–**d** young gametocyte in mononuclear leukocyte (**b**), macrogametocyte (**c**) and microgametocyte (**d**). Long simple arrows—parasites. Short simple arrows—parasite nuclei. Arrowheads—host cell nuclei. Giemsa-stained thin blood films

Haemosporidian parasites belonging to the genera *Plasmodium*, *Haemoproteus* and *Leucocytozoon* were reported (Tables 1, 2, Fig. 3). The overall prevalence of haemosporidians in birds maintained in open aviaries was 23.1% (24/104), as determined by PCR-based diagnostics. However, this test positive rate was approximately seven-fold greater in juveniles in comparison with adults (Table 1), indicating marked susceptibility of chicks and active local transmission. Furthermore, death rates of infected birds maintained in open aviaries was 7.7% (8/104). Post-mortem examination revealed characteristic features of haemosporidian infection, particularly the purple colour and hyperplasia of liver, which contained numerous randomly distributed red-to-black foci and calcifications, the enlarged black spleen, and the swollen reddened lungs (Additional file 1). Bacterial, viral and other pathogens were not reported in dead cranes.

**Table 2:** Haemosporidian parasites and mortality rate reported in infected juvenile cranes maintained in open aviaries in Beijing Zoo, 2007–2014

Bird species	No infected/No died	Parasite reported by PCR-based testing	Parasite reported by microscopic examination
*Anthropodies paradisea*	1/1	pANTPAR01 [1]^b^	*Leucocytozoon* sp. [1]
*Bugeranus carunculatus*	1/1	pANTPAR01 [1]	*Leucocytozoon* sp. [1]
*Grus japonensis*	4/2	pSGS1 [2]	*Leucocytozoon* sp. [2]
*Grus nigricollis*	9/4	pSGS1 [3], pGRUNIG01 [1]	*Leucocytozoon* sp. [4]
Total (4 species)	15/8 (53.3)^a^	4 lineages	One unidentified morphotype

Lineages of *Plasmodium* parasites were reported in all died cranes, but DNA of *Leucocytozoon* parasites was not amplified from samples, in which gametocytes of leucocytozoids were readily visible in blood films. Other symbols are as in Table 1

^a^Percentage of died birds is given in parentheses

^b^Number of reported infections is given in brackets

Twenty-five PCR-positive samples were sequenced. Eight lineages of haemosporidian parasites were reported. Among them, there were 5 lineages of *Plasmodium* parasites (*Plasmodium relictum* pSGS1, *Plasmodium homonucleophilum* pSW2 and 3 lineages of morphologically unidentified malaria parasites), one lineage of *Haemoproteus antigonis* (hGRUVIP01), and 2 lineages of *Leucocytozoon* (*Leucocytozoon majoris* lCB1 and one lineage lGRUJAP02 of unidentified parasites) (Table 1).

Malaria parasites of the genus *Plasmodium* (Fig. 3a) were rare in adults (1 case reported), but predominated in juveniles, in which these parasites represented 95% of all PCR-based reported haemosporidian infections. Prevalence of *Plasmodium* species in juveniles was approximately 28-fold greater than in adults (Table 1). *Haemoproteus antigonis* (hGRUVIP01) was found only as single infections in 1 adult and 1 juvenile white-naped crane (Table 1). *Leucocytozoon* sp. (probably *Leucocytozoon majoris*, the lineage lCB1) infection was detected in one White-naped crane. A morphologically unidentified lineage of *Leucocytozoon* sp. was detected in one adult Red-crowned crane (Table 1 and Fig. 3b–d).

The majority of reported PCR-based diagnostics were single infections (Table 1). Co-infections were found in 1 White-napped crane by PCR testing; it was the co-infection of *Plasmodium homonucleophilum* (pSW2) and *Leucocytozoon majoris* (lCB1).

With the exception of *Haemoproteus antigonis* (hGRUVIP01), all lineages detected in adult and juvenile cranes were different (Table 1). There was only one lineage (GRUVIP01), which was found both in adult and juvenile cranes. Other reported lineages were pSW2, lGRUJAP02 and lCB1 in adult birds, and pSGS1, pANTPAR01, pGRUMON01 and pGRUNIG01 in juvenile birds. This suggests that juvenile birds gained haemosporidian infections from zoo birds other than crane-parents. The majority of lineages, with the exception of *Plasmodium relictum* pSGS1 and the lineage pGRUNIG01 (identical to the lineage pDENVID02, see http://mbio-serv2.mbioekol.lu.se/Malavi), were reported in cranes for the first time.

*Leucocytozoon* species gametocytes were common in blood films of eight juvenile cranes belonging to four species that died due to haemosporidiosis at the age between 20 days to 4 months (Fig. 3b–d). All these chicks were also PCR-positive for various *Plasmodium* infections (Table 2). In other words, the used PCR-based molecular diagnostics was insensitive in detection of *Leucocytozoon* infections in died juvenile cranes. Mortality was not reported in birds older than 4 months.

Molecular analysis revealed parasites belonging to three genera of haemosporidians (Fig. 2). The three reported novel lineages of haemosporidians were respectively clustered with their genetically most similar lineages of corresponding parasite genera.

Based on 100% genetic similarity, our lineage pGRUNIG01 belongs to *Plasmodium elongatum* (pGRW6) (Fig. 2). The scenario is the same with the lineage of *Plasmodium* sp. pANTPAR01 and *Plasmodium relictum* (pSGS1) (Fig. 2), as well as *Plasmodium* sp. pGRUMON01 and *Plasmodium circumflexum* (pTURDUS1).

Juvenile birds maintained in the greenhouse were free from haemosporidian parasites, but juveniles from the Crane Island were infected and some of them died during the same time periods. No mortality was reported in juveniles from the greenhouse. At age of 6 months, they were placed in open air aviaries, and survived till adult age.

## Discussion

Haemosporidian parasites have been insufficiently investigated in cranes (*Gruidae*). Two species of *Haemoproteus* (*Haemoproteus antigonis* and *Haemoproteus balearicae*), one species of *Leucocytozoon* (*Leucocytozoon grusi*) have been described from birds belonging to this family [1]. Species of *Plasmodium* have been found in cranes occasionally, but the majority of reports remained unidentified to parasite species levels [16, 31–33, 40]. This study documented presence of *Plasmodium relictum* (pSGS1) and *Plasmodium homonucleophilum* (pSW2) in cranes and also detected three lineages of morphologically unidentified malaria parasites infecting these birds. DNA sequences of these parasites were deposited to NCBI and can be used for malaria diagnostics (Fig. 2).

It is probable that the lineage pGRUNIG01 belongs to *Plasmodium elongatum*, a cosmopolitan virulent malaria parasite, which causes disease and even mortality in many bird species belonging to different orders [41–43]. However, blood stages of *Plasmodium elongatum* were not seen during this study, indicating that infection might kill birds on tissue stages, which destroyed stem bone marrow cells [1, 43]. This parasite and also the most prevalent and invasive *Plasmodium relictum* (Table 1), worth particular attention in programmes aiming the sustained ex situ conservation of cranes.

*Haemoproteus antigonis* was originally described in India [44], and it was reported for the first time in China during this study. Gametocytes of this parasite were not seen in the PCR positive birds (Table 1), but the reported DNA sequence (445 bp, GenBank accession MG980617) coincided with the sequence detected in cranes in the USA (616 bp, GenBank accession KX223873.1 [45]). Therefore, the reported parasite likely belongs to *Haemoproteus antigonis*. Phylogenetic analysis placed cyt*b* lineage of this parasite to the clade of *Haemoproteus* parasites, but this lineage took a separate position in regard to species belonging to subgenera *Parahaemoproteus* and *Haemoproteus* (Fig. 2). Because phylogenies based on *cytb* gene indicates vectors of haemosporidians [46, 47], it is possible that *Haemoproteus antigonis* might be transmitted by dipteran insects different from louse flies (vectors of species belonging to subgenus *Haemoproteus*) and *Culicoides* biting midges (vectors of *Parahaemoproteus* species) [45, 48]. Further field observations combined with experimental studies are needed to test this hypothesis. *Haemoproteus antigonis* was reported in 1 adult and 1 juvenile *Grus vipio*, indicating that transmission might occur locally and chicks gain infection from parents.

Parasites were not observed in blood films in the majority of PCR-positive samples (Table 1), indicating possible light parasitaemia or abortive infections, or both. The abortive haemosporidian infections on exo-erythrocytic stage have been described in birds, but remain insufficiently investigated [27, 43]. In contrast, the molecular diagnostics did not reveal *Leucocytozoon* infections in juvenile cranes. However, these birds were positive for leucocytozoids during microscopic examination of blood films, and numerous gametocytes of *Leucocytozoon* sp. on different stages of development were seen in blood films (Fig. 3b–d), indicating that the used PCR-based protocol did not amplify DNA of these crane parasites.

The recorded gametocytes of *Leucocytozoon* parasite (Fig. 3b–d) might belong to *Leucocytozoon grusi*, but additional morphological data and molecular studies are needed for final conclusion and development of molecular characterization of this infection. It is worth to note that lineage lCB1 of *Leucocytozoon majoris* was detected in one adult *Grus vipio.* This is a common parasite of passeriform birds [2], and it has been not reported in cranes or other non-passeriform birds before. Because gametocytes of *Leucocytozoon majoris* were not seen in blood films, it might be that a case of abortive infection of *Leucocytozoon majoris* on sporozoite or tissue stages was reported in cranes [49], which is worth for additional investigation due to possible high virulence of abortive infection in non-competent avian hosts [27].

Molecular characterization of three lineages, which were reported during this study, has been developed formerly. These are *Plasmodium relictum* pSGS1 [43], *Plasmodium homonucleophilum* pSW2 [50], and *Leucocytozoon majoris* lCB1 [51]. Identical *cytb* sequences were previously reported in Beijing Zoo cranes. Several haemosporidian parasite lineages have been detected in cranes in the past, and they were also present in other bird species. For example, the lineage pGRUNIG01 (identical to the lineage pDENVID02, GenBank accession KU057965.1) of *Plasmodium* sp., which was detected in one Black crowned crane *Balearica pavonina* in this study, was reported in the White-faced whistling duck *Dendrocygna viduata* in Brazil [25]. This indicates broad geographical distribution of this infection, which belongs to *Plasmodium elongatum* (Fig. 2). Interestingly, the lineage pSGS1 of *Plasmodium relictum* was the most prevalent lineage in this study (found in 15 bird individuals), but it was reported only once in cranes before [52]. The latter parasite is generalist, and it has broad host and geographic distribution [7, 53]. This study shows that *Plasmodium relictum* (pSGS1) often infects naive juvenile cranes, and probably kills them on tissue stages before development of parasitaemia because blood stages were not observed in blood films in died PCR-positive cranes (Table 2).

The high prevalence of malaria parasites in juvenile cranes indicates their high susceptibility (Table 1). Because lineages of malaria parasites detected in adult and juvenile birds were different, it was concluded that chicks likely gain infection from other zoo birds. In other words, adult cranes likely are not ‘guilty’ in infection of their chicks in the Beijing Zoo. It is also possible that these infections might abort their development on tissue stages because parasitaemia was reported only in a few individual cranes [27]. Transmission of haemosporidian parasites by dipteran vectors and mortality of various bird species due to *Plasmodium* and *Haemoproteus* infections has been documented in many zoos all over the world [8, 25], but remains insufficiently investigated in Beijing Zoo. Further studies on haemosporidian parasite diversity, tissue pathology and vectors are needed to better understand epidemiology of haemosporidiosis and significance of this disease in the zoo.

It is worth to note that gametocytes of *Leucocytozoon* parasites were seen in blood films in many dead juvenile cranes (Table 2), but the authors were unable to detect DNA sequences of these parasites. It seems that the reported leucocytozoids are virulent and, together with malaria parasites, are highly responsible for chick mortality. Several recent molecular studies reported insufficiency of broadly used PCR-based protocols in detection of haemosporidian infections that are visible in same blood samples microscopically [54–56]. Development of new primers for amplification of DNA of leucocytozoids in cranes and other non-passerine birds is an important task for current studies on avian haemosporidian parasites.

Naive birds often suffer due to parasitic infections at their first exposure, and even mortality might occur, particularly among juveniles [2, 3, 42, 57]. Larger bare skins of young cranes make them more easily accessible to vectors [58]. High prevalence of haemosporidians in juveniles (Table 1) is likely due to their naive immune status. Because of high mortality among juvenile cranes due to haemosporidian infections, the authors advise conservation projects to maintain chick until the age of approximately 6 months in vector-free aviaries. This study shows that maturing juvenile birds (approximately after 6-months) and adult cranes do not develop diseases from haemosporidian infections, survive and can be maintained in open-air aviaries and released in nature.

## Conclusion

The 8-year observations in Beijing Zoo show that juvenile cranes are markedly susceptible to *Plasmodium* and *Leucocytozoon* infections, which are gained from non-parent wild birds living in the zoo. Mortality is high in crane chicks. Because parasitaemia was not observed in the majority of studied cranes, the authors call for investigation of possible abortive haemosporidian infections, which might kill these birds during exoerythrocytic development before development of parasitaemia. Molecular diagnostics of crane *Leucocytozoon* parasites needs improvement. Adult cranes can resist haemosporidian parasites, which were commonly observed in juveniles. This study shows that haemosporidian infection can be readily prevented in chicks if these birds are maintained in vector-free aviaries until the 6-months age.

## Additional file

**Additional file 1.** Gross necropsy examination of a dead crane (*Grus nigricollis*, 10 weeks old) showed characteristic pathological features of haemosporidian infection. (A) Livers were tan to purple with hyperplasia and numerous, randomly distributed red-to-black foci pathological changes, ranging in size from 1 to 4 mm (blue arrows) and calcification spots with the white foci, ranging in size from 2 to 6 mm (yellow arrows). (B) Spleens was enlarged with the tense capsule and showed soft and friable consistency and coloured black hue. (C) Lungs showed swollen, reddened appearance and doughy consistency. (D) Slightly enlarged kidneys were found. (E) Heart failure after long course of disease. (F) Numerous gametocytes of *Leucocytozoon* sp. (arrows) in histological section of livers. These parasites were also numerous in spleen, lungs, kidneys and heart. Scale bar = 20 μm.

## References

[1] Valkiūnas G (2005). Avian malaria parasites and other *Haemosporidia*.

[2] Atkinson C, Atkinson CT, Thomas NJ, Hunter B (2008). Avian malaria. Parasitic diseases of wild birds.

[3] Marzal A, García-Longoria L, Cardenas Callirgos JM, Sehgal RNM (2015). Invasive avian malaria as an emerging parasitic disease in native birds of Peru. Biol Invasions.

[4] Perkins SL (2014). Malaria’s many mates: past, present, and future of the systematics of the order *Haemosporida*. J Parasitol.

[5] Pacheco MA, Matta NE, Valkiunas G, Parker PG, Mello B, Stanley CE (2018). Mode and rate of evolution of Haemosporidian mitochondrial genomes: timing the radiation of avian parasites. Mol Biol Evol.

[6] Sehgal RN (2015). Manifold habitat effects on the prevalence and diversity of avian blood parasites. Int J Parasitol Parasites Wildl..

[7] Bensch S, Hellgren O, Perez-Tris J (2009). MalAvi: a public database of malaria parasites and related haemosporidians in avian hosts based on mitochondrial cytochrome b lineages. Mol Ecol Resour..

[8] Ejiri H, Sato Y, Kim KS (2011). Blood meal identification and prevalence of avian malaria parasite in mosquitoes collected at Kushiro wetland, a subarctic zone of Japan. J Med Entomol.

[9] Outlaw DC, Ricklefs RE (2014). Species limits in avian malaria parasites (*Haemosporida*): how to move forward in the molecular era. Parasitology.

[10] Clark NJ, Clegg SM, Lima MR (2014). A review of global diversity in avian haemosporidians (*Plasmodium* and *Haemoproteus*: *Haemosporida*): new insights from molecular data. Int J Parasitol.

[11] Scheuerlein A, Ricklefs RE (2004). Prevalence of blood parasites in European passeriform birds. Proc Biol Sci..

[12] Schrader MS, Walters EL, James FC, Greiner EC (2003). Seasonal prevalence of a haematozoan parasite of red-bellied woodpeckers (*Melanerpes carolinus*) and its association with host condition and overwinter survival. The Auk.

[13] Woodworth BL, Atkinson CT, LaPointe DA, Hart PJ, Spiegel CS (2005). Host population persistence in the face of introduced vector-borne diseases: Hawaii amakihi and avian malaria. Proc Natl Acad Sci USA.

[14] Ricklefs RE, Medeiros M, Ellis VA, Sevensson-Coelho M, Blake JG, Loiselle BA (2016). Avian migration and the distribution of malaria parasites in New World passerine birds. J Biogeogr.

[15] Inumaru M, Murata K, Sato Y (2017). Prevalence of avian *haemosporidia* among injured wild birds in Tokyo and environs, Japan. Int J Parasitol Parasites Wildl..

[16] Scott HH (1926). Report on the deaths occurring in the Society’s Gardens during the year 1925. Proc Zool Soc London.

[17] Scott HH (1927). Report on the deaths occurring in the Society’s Gardens during the Year 1926. Proc Zool Soc London.

[18] Fix AS, Waterhouse C, Greiner EC, Stoskopf MK (1988). *Plasmodium relictum* as a cause of avian malaria in wild-caught Magellanic penguins (*Spheniscus magellanicus*). J Wildl Dis.

[19] Graczyk TK, Cranfield MR, McCutchan TF, Bicknese EJ (1994). Characteristics of naturally acquired avian malaria infections in naive juvenile African black-footed penguins (*Spheniscus demersus*). Parasitol Res.

[20] Belo NO, Passos LF, Júnior LM, Goulart CE, Sherlock TM, Braga EM (2009). Avian malaria in captive psittacine birds: detection by microscopy and 18 s rRNA gene amplification. Prev Vet Med..

[21] Baron HR, Howe L, Varsani A, Doneley RJ (2014). Disease screening of three breeding populations of adult exhibition budgerigars (*Melopsittacus undulatus*) in New Zealand reveals a high prevalence of a novel polyomavirus and avian malaria infection. Avian Dis.

[22] Griner L, Sheridan B (1967). Malaria (*Plasmodium relictum*) in penguins at the San Diego Zoo. Vet Clin Pathol..

[23] Martinez-de LPJ, Muñoz J, Capelli G, Montarsi F, Soriguer R, Arnoldi D (2015). Avian malaria parasites in the last supper: identifying encounters between parasites and the invasive Asian mosquito tiger and native mosquito species in Italy. Malar J..

[24] Vanstreels RE, Kolesnikovas CK, Sandri S, Silveira P, Belo NO, Ferreira Junior FC (2014). Outbreak of avian malaria associated to multiple species of *Plasmodium* in Magellanic penguins undergoing rehabilitation in southern Brazil. PLoS ONE.

[25] Chagas CR, Guimarães Lde O, Monteiro EF, Valkiūnas G, Katayama MV, Santos SV (2016). *Hemosporidian* parasites of free-living birds in the Sao Paulo Zoo, Brazil. Parasitol Res..

[26] King RS, McKann PC, Gray BR, Putnam MS (2015). Host-parasite behavioral interactions in a recently introduced, whooping crane population. J Fish Wildl Manag..

[27] Valkiūnas G, Iezhova TA (2017). Exo-erythrocytic development of avian malaria and related haemosporidian parasites. Malar J..

[28] Cao M, Xu H, Le Z, Zhu M, Cao Y (2015). A multi-scale approach to investigating the red-crowned crane-habitat relationship in the Yellow River Dalta Nature Reserve, China: implication for conservation. PLoS ONE.

[29] Fakarayi T, Mashapa C, Gandiwa E, Kativu S (2016). Varying land-use has an influence on wattled and grey crowned cranes’ abundance and distribution in Driefontein Grasslands important bird area, Zimbabwe. PLoS One..

[30] Zhang L, An B, Shu M, Yang X (2017). Nest-site selection, reproductive ecology and shifts within core-use areas of black-necked cranes at the northern limit of the Tibetan Plateau. PeerJ..

[31] Schwetz J (1944). Notes protozoologiques au le Congo belge. Ann Soc Belg Med Trop.

[32] Halpern N, Bennett GF (1983). *Haemoproteus* and *Leucocytozoon* infections in birds of the Oklahoma City Zoo. J Wildl Dis.

[33] Shimizu T, Yasuda N, Kono I, Hanada K (1984). Avian malaria-like disease found in cranes (*Grus monacha*). Memoirs Faculty Agriculture Kagoshima Univ..

[34] Jia T, Zhang C, Liu X, Yang M, Hao L, Hao L (2010). PCR determination of Haemosporidian parasites, infecting juvenile red-crowned crane in Beijing Zoo. Chin J Wildl..

[35] Hellgren O, Waldenstrom J, Bensch S (2004). A new PCR assay for simultaneous studies of *Leucocytozoon*, *Plasmodium*, and *Haemoproteus* from avian blood. J Parasitol.

[36] Darriba D, Taboada GL, Doallo R, Posada D (2012). jModelTest 2: more models, new heuristics and parallel computing. Nat Methods.

[37] Drummond AJ, Rambaut A (2007). BEAST: Bayesian evolutionary analysis by sampling trees. BMC Evol Biol.

[38] Perez-Tris J, Bensch S (2005). Diagnosing genetically diverse avian malarial infections using mixed-sequence analysis and TA-cloning. Parasitology.

[39] Kumar S, Stecher G, Tamura K (2016). MEGA7: Molecular evolutionary genetics analysis version 7.0 for bigger datasets. Mol Biol Evol.

[40] Hamerton BCAE (1941). Report on the deaths occurring in the Society’s Gardens during the Years 1939–1940. Proc Zool Soc London.

[41] Valkiūnas G, Zehtindjiev P, Dimitrov D, Krizanauskiene A, Iezhova TA, Bensch S (2008). Polymerase chain reaction-based identification of *Plasmodium* (*Huffia*) *elongatum*, with remarks on species identity of haemosporidian lineages deposited in GenBank. Parasitol Res.

[42] Howe L, Castro IC, Schoener ER, Hunter S, Barraclough RK, Alley MR (2012). Malaria parasites (*Plasmodium* spp.) infecting introduced, native and endemic New Zealand birds. Parasitol Res.

[43] Palinauskas V, Žiegytė R, Iezhova TA, Ilgūnas M, Bernotienė R, Valkiūnas G (2016). Description, molecular characterisation, diagnostics and life cycle of *Plasmodium elongatum* (lineage pERIRUB01), the virulent avian malaria parasite. Int J Parasitol.

[44] De Mello IF. New haemoproteids of some Indian birds. In: Proc Indian Acad Sci Sect (Sec. B). 1935;2:469–475.

[45] Bertram MR, Hamer SA, Hartup BK, Snowden KF, Medeiros MC, Outlaw DC (2017). A novel *Haemosporida* clade at the rank of genus in North American cranes (*Aves*: *Gruiformes*). Mol Phylogenet Evol.

[46] Bukauskaite D, Žiegytė R, Palinauskas V, Iezhova TA, Dimitrov D, Ilgūnas M (2015). Biting midges (*Culicoides*, *Diptera*) transmit Haemoproteus parasites of owls: evidence from sporogony and molecular phylogeny. Parasit Vectors..

[47] Žiegytė R, Markovets MY, Bernotienė R, Mukhin A, Iezhova TA, Valkiūnas G (2017). The widespread biting midge *Culicoides impunctatus* (*Ceratopogonidae*) is susceptible to infection with numerous *Haemoproteus* (*Haemoproteidae*) species. Parasit Vectors..

[48] Santiago-Alarcon D, Palinauskas V, Schaefer HM (2012). Diptera vectors of avian Haemosporidian parasites: untangling parasite life cycles and their taxonomy. Biol Rev Camb Philos Soc.

[49] Valkiūnas G, Iezhova TA, Loiseau C, Sehgal RN (2009). Nested cytochrome B polymerase chain reaction diagnostics detect sporozoites of hemosporidian parasites in peripheral blood of naturally infected birds. J Parasitol.

[50] Ilgūnas M, Palinauskas V, Iezhova TA, Valkiūnas G (2013). Molecular and morphological characterization of two avian malaria parasites (*Haemosporida*: *Plasmodiidae*), with description of *Plasmodium homonucleophilum* n. sp.. Zootaxa..

[51] Perkins SL (2008). Molecular systematics of the three mitochondrial protein-coding genes of malaria parasites: corroborative and new evidence for the origins of human malaria. Mitochondrial DNA..

[52] Waldenström J, Bensch S, Kiboi S, Hasselquist D, Ottosson U (2002). Cross-species infection of blood parasites between resident and migratory songbirds in Africa. Mol Ecol.

[53] Beadell JS, Gering E, Austin J, Dumbacher JP, Peirce MA, Pratt TK (2004). Prevalence and differential host-specificity of two avian blood parasite genera in the Australo-Papuan region. Mol Ecol.

[54] Zehtindjiev P, Križanauskienė A, Bensch S, Palinauskas V (2012). A new morphologically distinct avian malaria parasite that fails detection by established polymerase chain reaction-based protocols for amplification of the cytochrome B gene. J Parasitol.

[55] Valkiūnas G, Ilgūnas M, Bukauskaitė D, Iezhova TA (2016). Description of *Haemoproteus ciconiae* sp. nov. (*Haemoproteidae*, *Haemosporida*) from the white stork *Ciconia ciconia*, with remarks on insensitivity of established polymerase chain reaction assays to detect this infection. Parasitol Res.

[56] Schaer J, Reeder DM, Vodzak ME, Olival KJ, Weber N, Mayer F (2015). *Nycteria* parasites of Afrotropical insectivorous bats. Int J Parasitol.

[57] Bueno MG, Lopez RP, de Menezes RM, de Costa-Nascimento MJ, Lima GF, Araujo RA (2010). Identification of *Plasmodium relictum* causing mortality in penguins (*Spheniscus magellanicus*) from Sao Paulo Zoo, Brazil. Vet Parasitol..

[58] Imura T, Suzuki Y, Ejiri H, Sato Y, Ishida K, Sumiyama D (2012). Prevalence of avian *haematozoa* in wild birds in a high-altitude forest in Japan. Vet Parasitol.

