# Supplementary figures and images for: Malaria parasites and related haemosporidians cause mortality in cranes: a study on the parasites diversity, prevalence and distribution in Beijing Zoo
Source: Malar J. 2018 Jun 18;17:234. doi: 10.1186/s12936-018-2385-3 (PMC6006844; doi:10.1186/s12936-018-2385-3)

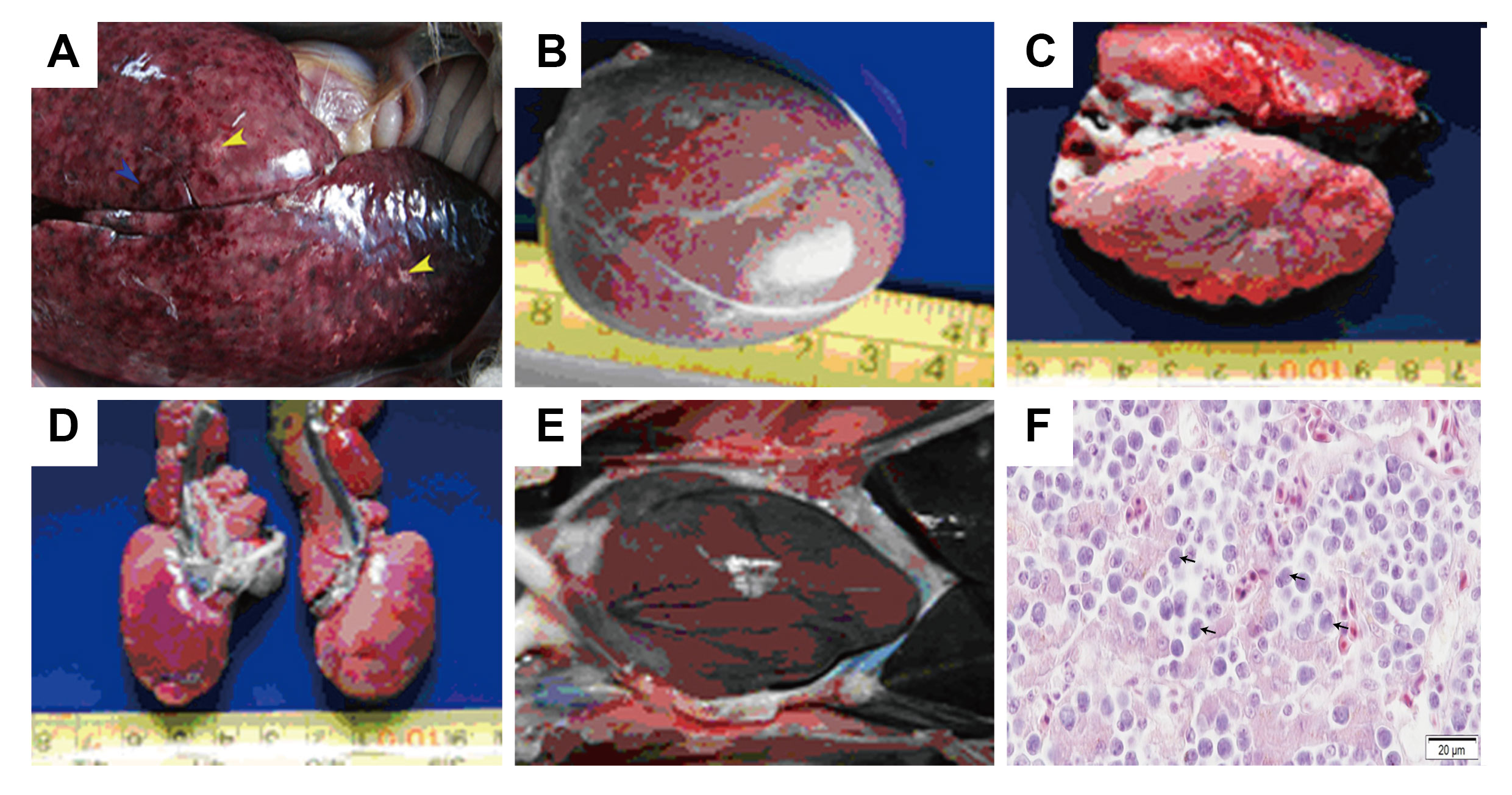

Supplement: Supplementary file 1 — Additional file 1. Gross necropsy examination of a dead crane (Grus nigricollis, 10 weeks old) showed characteristic pathological features of haemosporidian infection. (A) Livers were tan to purple with hyperplasia and numerous, randomly distributed red-to-black foci pathological changes, ranging in size from 1 to 4 mm (blue arrows) and calcification spots with the white foci, ranging in size from 2 to 6 mm (yellow arrows). (B) Spleens was enlarged with the tense capsule and showed soft and friable consistency and coloured black hue. (C) Lungs showed swollen, reddened appearance and doughy consistency. (D) Slightly enlarged kidneys were found. (E) Heart failure after long course of disease. (F) Numerous gametocytes of Leucocytozoon sp. (arrows) in histological section of livers. These parasites were also numerous in spleen, lungs, kidneys and heart. Scale bar = 20 μm. [file 12936_2018_2385_MOESM1_ESM.jpg]
